# Quantification of Plasmid Copy Number with Single Colour Droplet Digital PCR

**DOI:** 10.1371/journal.pone.0169846

**Published:** 2017-01-13

**Authors:** Magdalena Plotka, Mateusz Wozniak, Tadeusz Kaczorowski

**Affiliations:** Laboratory of Extremophiles Biology, Department of Microbiology, University of Gdansk, Gdansk, Poland; University of Hyogo, JAPAN

## Abstract

Bacteria can be considered as biological nanofactories that manufacture a cornucopia of bioproducts most notably recombinant proteins. As such, they must perfectly match with appropriate plasmid vectors to ensure successful overexpression of target genes. Among many parameters that correlate positively with protein productivity plasmid copy number plays pivotal role. Therefore, development of new and more accurate methods to assess this critical parameter will result in optimization of expression of plasmid-encoded genes. In this study, we present a simple and highly accurate method for quantifying plasmid copy number utilizing an EvaGreen single colour, droplet digital PCR. We demonstrate the effectiveness of this method by examining the copy number of the pBR322 vector within *Escherichia coli* DH5α cells. The obtained results were successfully validated by real-time PCR. However, we observed a strong dependency of the plasmid copy number on the method chosen for isolation of the total DNA. We found that application of silica-membrane-based columns for DNA purification or DNA isolation with use of bead-beating, a mechanical cell disruption lead to determination of an average of 20.5 or 7.3 plasmid copies per chromosome, respectively. We found that recovery of the chromosomal DNA from purification columns was less efficient than plasmid DNA (46.5 ± 1.9% and 87.4 ± 5.5%, respectively) which may lead to observed differences in plasmid copy number. Besides, the plasmid copy number variations dependent on DNA template isolation method, we found that droplet digital PCR is a very convenient method for measuring bacterial plasmid content. Careful determination of plasmid copy number is essential for better understanding and optimization of recombinant proteins production process. Droplet digital PCR is a very precise method that allows performing thousands of individual PCR reactions in a single tube. The ddPCR does not depend on running standard curves and is a straightforward and reliable method to quantify the plasmid copy number. Therefore we believe that the ddPCR designed in this study will be widely used for any plasmid copy number calculation in the future.

## Introduction

Plasmids play an important role in molecular biology and biotechnology, primarily as vectors for molecular cloning to facilitate the overproduction of recombinant proteins [1], but also as sophisticated nanotools for specialized applications in the genome engineering [2]. In a rapidly growing field of gene therapy and genetic vaccination, naked or lipid-coated plasmid DNA is also successfully applied to administer therapeutic genes [3] and is considered to be much safer and easier to use than genetically modified viruses [4, 5]. Moreover, plasmid-oriented studies provide insights to improve understanding of DNA replication, maintenance and transfer strategies which are essential to all microorganisms [6, 7]. In this respect, among many features that characterize these mobile genetic elements, the one which defines the number of plasmid units that are contained inside one bacterial cell is especially important, both from a practical and a biological point of view.

Plasmid copy number (PCN) determines the gene dosage which is defined theoretically as number of genetic units accessible for expression. Therefore, quantification of the plasmid copy number is crucial in describing an expression system and exerts strong impact on protein production [8]. Generally, high-copy plasmids are preferred for efficient overproduction of recombinant proteins that do not affect the host viability, but in case of toxic or unstable proteins, usually low-copy plasmids are used [1].

Numerous methods that have been developed for calculating the plasmid copy number can be divided into two main categories: the direct and the indirect methods. The latter include the correlation of plasmid copy number with the activity of an enzyme/protein coded on the plasmid [8]. The examples include β-lactamase, luciferase or green fluorescent reporter protein [9–11]. These methods are prone to errors because the activity of such enzyme/protein, except for their dependence on PCN, also relies on such factors as the mRNA stability, proteolysis and protein folding, and these may vary significantly [8]. The direct methods include: (*i*) agarose gel electrophoresis followed by densitometry [12, 13], (*ii*) capillary electrophoresis [14], (*iii*) CsCl or ethidium bromide-CsCl gradient centrifugation [15], (*iv*) high-performance liquid chromatography (HPLC) [16], and techniques based on radiolabelling of the nucleic acids [17]. All of these techniques are either complicated, time-consuming, have poor reproducibility or can cause safety problems with handling of radioactively labelled probes [13, 18]. Therefore, there is a constant need for new and more accurate methods to determine the plasmid copy number.

Development of real-time polymerase chain reaction (qPCR) for the detection and quantification of nucleic acids opened a gate for application of qPCR for PCN determination in bacteria [19]. To calculate the PCN, two single copy genes: one of the bacterial chromosome (reference gene) and one of a plasmid (target gene) need to be amplified. The calculated copy ratio of the target gene to the reference gene defines the PCN [20]. Two sensitive and precise calculation methods: absolute and relative, are available for quantification of PCN by qPCR [21]. However, both calculations rely on the presence of the external standards. Absolute quantification determines the target gene copy number by relating the PCR signal to a standard curve. Relative quantification (ΔΔC_T_ method) presents the amount of the target gene in relation to the plasmid (calibrator), where both the target and the reference genes are at a constant ratio of 1:1 [20, 22]. In summary, the accuracy of the absolute and relative qPCR quantification, despite such factors as the sample preparation and the choice of a reference gene, largely depends on the quality of the standard [19], and this is the main drawback of this method.

Recently, droplet digital PCR (ddPCR) emerged as a new technology that enables processing of ~20,000 up to 1,000,000 separate PCR reactions in a single tube [23, 24]. This method facilitates the accurate and precise quantitation of nucleic acid targets without the need for calibration curves and any external standards [23]. By using a droplet generator, it is possible to partition a fluorescent-dye based PCR assay into a highly uniform, less than one-nanoliter-volume, water-in-oil droplets. Conventional PCR amplification is then performed in a thermal cycler in 96-well PCR plates. The end-point measurement of the nucleic acid quantity is performed by placing the plate in a ddPCR droplet reader. The reader sips droplets from each well and streams them in a single-file past a two-colour fluorescence detector at the rate of ~1,500 droplets per second. Droplets are annotated as positive or negative based on their fluorescence amplitude, and the number of positive and negative signals is utilized to calculate the concentration of the target DNA with use of Poisson statistics [23, 25]. The ddPCR allows to perform either a duplex fluorescent-probe-based PCR assay or, more recently, a single colour fluorescent PCR with the use of a nonspecific DNA binding dye (EvaGreen). The technology based on intercalation of the double-stranded DNA-binding dyes, such as EvaGreen, is simple and inexpensive, and does not need any additional fluorescent-labelled probes [26]. By differentiating the length of the target and the reference amplicons, it is also possible to distinguish between their fluorescent signals and quantify each of them independently in the same tube.

Here, we demonstrate the effectiveness of the ddPCR technology in determination of the PCN in bacteria. In our report, we used as a model *E*. *coli* cells carrying the plasmid pBR322 [27]. We verified the accuracy of the novel digital methodology by comparing the copy number calculations with the data obtained by real-time PCR. Moreover, we have shown that the DNA extraction method (the commercial total DNA isolation kit *vs* mechanical cell disruption) can affect the PCN assessment, as well as that this parameter depends on bacterial growth phase and bacterial culture media used. We strongly believe that single colour, droplet digital PCR developed in this study can be used universally for the PCN determination of any plasmid.

## Materials and Methods

### Strains, plasmids and DNA isolation procedures

*E*. *coli* DH5α [pBR322] cells were cultured in (*i*) Luria-Bertani (LB) medium (SIGMA Aldrich), (*ii*) Terrific Broth (TB), comprised of per liter: 12.0 g Tryptone, 24.0 g Yeast Extract, 9.4 g K_2_HPO_4_, 2.2 g KH_2_PO_4_, pH 7.2 ± 0.2 at 25°C [28], (*iii*) M9 minimal medium, comprised of per liter: 6.8 g Na_2_HPO_4_, 3.0 g KH_2_PO_4_, 0.5 g NaCl, 1.0 g NH_4_Cl, 2 mM MgSO_4_, 0.1 mM CaCl_2_, 1 mM thiamine hydrochloride, and 0.2% (w/v) glucose, pH 7.4 [29] at 37°C with addition of 100 μg/ml of ampicillin (SIGMA Aldrich). At the mid-logarithmic growth phase (OD_600_ of 0.5) or stationary growth phase (OD_600_ of 1.5, when indicated), 1-ml aliquots were removed from the culture and the bacteria were harvested by centrifugation. The total DNA was isolated either with the use of QIAamp DNA Mini Kit (QIA; Qiagen), following the protocol for bacterial cells (1 h lysis at 56°C), or by the bead-beating (BB) method. For the latter procedure, the Hybaid RiboLyser (Hybaid, Teddington, UK) was used as a mechanical cell disruptor. The Zirconia/Silica (BioSpec Products) 1 mm beads (1.0 g) were placed in a screw-cap 2.0 ml sample tubes and bacteria suspended in buffer A (20 mM Tris-HCl pH 8.0, 50 mM NaCl, 0.1% Triton X-100) were added. Then, the samples were homogenized for 45 s at the 6.5 speed setting and centrifuged (5 min, 20,000 × g). The resulting supernatant, containing the *E*. *coli* total DNA, was aliquoted to avoid repeated freezing and thawing of the samples, frozen in liquid nitrogen and stored at– 70°C for further analysis. The total DNA concentration after isolation with QIAamp DNA Mini Kit was measured using a NanoDrop 1000 UV-VIS spectrophotometer (Thermo Scientific). The template DNA isolated with the use of the QIAamp DNA Mini Kit was normalized to 2 ng μl^-1^ and samples were stored frozen until further analysis. Vector pGEM3Zf(+) (Promega) was used for molecular cloning. Recombinant plasmid pGEM-dxs that was used as a calibrator, was constructed in this study and is deposited in the Collection of Plasmids and Microorganisms, University of Gdansk, Poland.

### Droplet digital PCR (ddPCR) assay conditions

Primers used in the droplet digital PCR were either used previously for *bla* and *dxs* qPCR amplification (Table 1; set A) [20] or designed in this study by using of Primer3Plus programme (Table 1B; set B). All primers were purchased form Genomed S.A. (Poland) and were designed to be 20 bp in length and to have the melting temperature within 2.1°C of each other. The usage of the set A primer pairs resulted in PCR product amplification with the length of 81 bp and 113 bp for *bla* and *dxs*, respectively. The length of the PCR products for multiplex reaction (primer set B) was design to be 69 bp and 160 bp for *bla* and *dxs*, respectively. For each primer pair, a simplex PCR was performed using *E*. *coli* DH5α [pBR322] total DNA as a template, followed by electrophoresis in 1.7% agarose gel to confirm the correct size of the product. For all 20 μl ddPCR reaction mixtures assembled, 2× EvaGreen ddPCR Supermix (Bio-Rad) and primers at a final concentration of 0.2 μM were included. No template controls (NTC) were used to monitor contaminations and primer-dimer formation. Reactions were equilibrated for 3 min at room temperature and dispensed into each well of the droplet generator DG8 cartridge (Bio-Rad). Each oil compartment of the cartridge was filled with 70 μl of droplet generation oil for EvaGreen (Bio-Rad), and approximately 20,000 droplets were generated at each well with use of the droplet generator (Bio-Rad QX200). The entire droplet emulsion volume (40 μl) was further loaded onto a 96-well PCR plate (Eppendorf). The plate was then heat sealed with a pierceable foil in the PX1 PCR Plate Sealer (Bio-Rad), and placed in a Mastercycler ep gradient S thermocycler (Eppendorf). The optimal thermal cycling conditions were used: 95°C for 5 min; 35 cycles of 95°C for 30 s, 58°C for 30 s, 72°C for 1 min; and a final step at 72°C for 1 min. The reaction mixtures were then held at 4°C until needed. The reactions were optimized with respect to the primer annealing step (in the temperature range between 50 and 62°C) and 58°C has been chosen as the annealing temperature for further analysis (data not shown). The cycled droplets were read individually with the QX200 droplet-reader (Bio-Rad), and analysed with QuantaSoft droplet reader software, version 1.6.6.0320 (Bio-Rad). The error reported for a single well was the Poisson 95% confidence interval.

**Table 1:** Specification of primers used in this study.

A	Sequences of primers used for real-time PCR and digital droplet PCR (set A)			
Target	Primers (5’→3’)	Length (nt)	Melting temperature (°C)	Product size (bp)
*bla*	Forward: CTACGATACGGGAGGGCTTA	20	53.8	
	Reverse: ATAAATCTGGAGCCGGTGAG	20	51.8	81
*dxs*	Forward: CGAGAAACTGGCGATCCTTA	20	51.8	
	Reverse: CTTCATCAAGCGGTTTCACA	20	49.7	113
B	Sequences of primers used for multiplex digital droplet PCR (set B)			
Target	Primers (5’→3’)	Length (nt)	Melting temperature (°C)	Product size (bp)
*bla*	Forward: GCACCTATCTCAGCGATCTG	20	53.8	
	Reverse: AGTTATCTACACGACGGGGA	20	51.8	69
*dxs*	Forward: GCTGGTCGATATGCGTTTTG	20	51.8	
	Reverse: GGGTACTGGTTTACGATGGG	20	53.8	160

### Construction of the standard curves for SYBR Green I qPCR

The D-1-deoxyxylulose 5-phosphate synthase gene (*dxs*, GenBank accession number AF035440) was amplified by PCR from the *E*. *coli* DH5α genomic DNA using forward dxs_F: 5’-GATCAAGCTTGATATCCTGAGTTCCTTGCGGAATAAAG -3’ and reverse dxs_R: 5’-CTAGAAGCTTCCGGTCCTGTTCG -3’ primer set (the HindIII site is underlined). 2 × PCR MasterMix was used for DNA amplification, according to the manufacturer's instructions (A&A Biotechnology). The PCR product (0.58-kb) was digested with HindIII and then ligated into pGEM-3Zf(+) (Promega) previously linearized with the same enzyme. The resulting construct (pGEM-dxs), was used to transform *E*. *coli* DH5α [30]. The DNA sequence of the recombinant clone was verified by sequencing. The pGEM-dxs plasmid contains two separate sequences, specific for β-lactamase (*bla*) and *dxs* target genes. The concentration of the pGEM-dxs was measured using Qubit dsDNA BR Assay Kit (Invitrogen), according to the manufacturer’s recommendations, with the use of Qubit 2.0 Fluorometer (Invitrogen). The theoretical plasmid copy number was calculated with the use of the ENDMEMO software: http://www.endmemo.com/bio/dnacopynum.php. Five 10-fold dilutions of the pGEM-dxs, ranging from 1 × 10^5^ to 1 × 10^9^ copies μl^-1^, were used to construct the standard curves for *bla* and *dxs*, respectively. Amplification of the target sequences (*dxs* and *bla*) was performed using previously published primer sets which are listed in Table 1A (Set A) [20]. Real-time PCR amplification and analysis were performed using a LightCycler 480 instrument with 1.5.0 software version (Roche Diagnostics). All real-time PCR runs were performed in triplicate, and each reaction mixture was prepared with the use of a LightCycler 480 SYBR Green I Master kit (Roche Diagnostics). The kit contains PCR-grade H_2_O and 2× concentrated Master mix (the mixture of FastStart Taq DNA Polymerase, reaction buffer, dNTP mix (with dUTP instead of dTTP), SYBR Green I dye, and MgCl_2_). The PCR reaction was performed in a total volume of 10 μl containing 3.0 μl of PCR-grade H_2_O, 5 μl of 2× concentrated Master mix, 0.5 μl of each primer (final concentration 0.5 μM), and 1 μl template DNA at specified concentrations indicated.

The following thermal cycling conditions were used: 95°C for 10 min; 40 cycles of 95°C for 10 s, 62°C for 10 s, 72°C for 10 s. The fluorescence signal was measured at the end of each extension step at 72°C. After amplification, a melting peak analysis with a temperature gradient of 0.1°C s^-1^, from 70 to 95°C, was performed to confirm that only the specific products were amplified. Finally, the samples were cooled down to 40°C for 30 s. Real-time PCR assays were optimized with respect to the MgCl_2_ concentration. We found that while no addition of MgCl_2_ was required for efficient amplification with use of the *bla*-primer set, in case of the *dxs* gene detection it was necessary to supplement the reaction mixture with MgCl_2_ to final concentration of 3 mM. Each standard curve was generated by the LightCycler480 SW 1.5 programme. The values of the threshold cycles (C_T_) for *bla* and *dxs* were determined by the “Fit Points Method” software. The C_T_ values were plotted against the logarithm of a theoretical copy number for each pGEM-dxs template dilution. Standard curves for *bla* and *dxs* were generated by a linear regression of the plotted points. For each standard curve, PCR amplification efficiency (E) was calculated by the programme according to the equation: E = 10^−1/slope^– 1 (100% efficiency = 1).

### Absolute and relative quantification by real-time PCR

Absolute and relative quantification methods were used to quantify the PCN [19, 20, 22]. Both, *bla* and *dxs* are single-copy genes of pBR322 and *E*. *coli* chromosomal DNA, respectively. Therefore, the plasmid copy number can be determined as the copy ratio of *bla* to *dxs*: PCN = [copy number of *bla*]/[copy number of *dxs*]. Absolute quantification calculates the copy number of *bla* and *dxs* genes by relating the PCR signal (C_T_ value) of a sample to the standard curve. A relative quantification presents the amount of the target gene (in our case the *bla* gene) relative to the *dxs* gene and to pGEM-dxs, which contains both, the target (*bla*) and the reference (*dxs*) sequences with a constant ratio of 1:1. The target/reference ratios of all samples were normalized by the target/reference ratio of the pGEM-dxs. For the ΔΔC_T_ calculation, the amplification efficiencies (E) of the target and the reference must be approximately equal [22]. The results are expressed as a fold ratio of the normalized *bla* gene amounts. The relative amount of *bla =* (1 + E) –^ΔΔCT^, where ΔΔC_T_ = ΔC_T_ of the sample (the total DNA extracted from *E*. *coli* [pBR322]) − ΔC_T_ of the calibrator (pGEM-dxs); ΔC_T_ = C_T_ of *bla* − C_T_ of *dxs*.

### Efficiency of chromosomal and plasmid DNA purification with QIAamp DNA mini columns

Chromosomal and plasmid DNA were isolated from *E*. *coli* DH5α and *E*. *coli* DH5α [pBR322] with the use of Genomic and Plasmid DNA mini kits, respectively (A&A Biotechnology). DNA concentrations were measured by NanoDrop 1000 UV-VIS spectrophotometer (Thermo Scientific). Genomic DNA in the following amounts: 2110 ng, 1855 ng and 3922 ng, were paired with 524 ng, 1100 ng and 1684 ng of plasmid DNA, respectively in three independent experiments. The suspensions of genomic and plasmid DNA were mixed separately with 100 μl of a lysis buffer (Qiagen). DNA isolations were performed using the QIAamp DNA Mini Kit following manufacturer’s instructions (Qiagen). Concentration of the eluted DNA was measured by ND-1000 Spectrophotometer and the ratio of the recovered to loaded DNA was calculated.

### Statistical analysis

The statistical significance was assessed by the two tailed Student unpaired *t* test with use of GraphPad Prism 5.0 software with a *P* value <0.05 (95% confidence interval).

## Results

### PCN determination by droplet digital PCR

**Fig 1:**
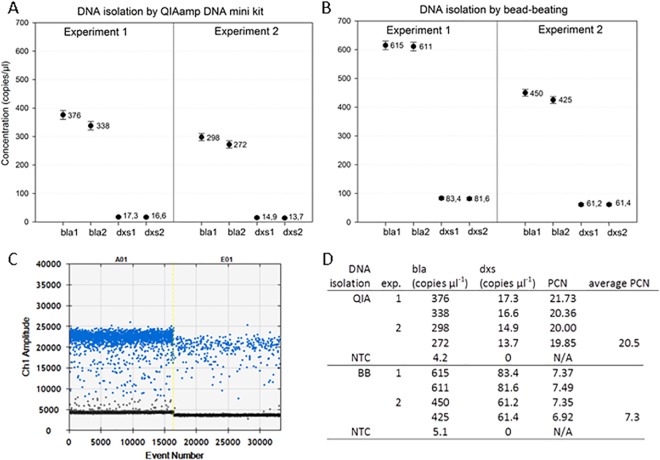
Quantification of pBR322 plasmid copy number by digital droplet PCR. *E*. *coli* DH5α total DNA isolated by the bead beating method (A) and the QIAamp DNA mini kit (B), from two independent bacterial cultures in a logarithmic growth phase (Experiment 1 and 2), served as a template for the *bla* and *dxs* ddPCR amplification with the use of primer set A (Table 1A). Each experiment was run in two replicates (bla1, bla2 and dxs1, dxs2). Error bars indicate the 95% confidence limits as determined from the Poisson distribution. (C) Columns A01 and E01 represents single wells of ~ 20,000 droplets after ddPCR amplification of *bla* and *dxs*, respectively. (D) Estimated pBR322 copy number by digital droplet PCR. The plasmid copy number of pBR322 was calculated by dividing the copy number of *bla* by the copy number of *dxs*. Average PCN from four measurements was determined to be 20.5 for QIA and 7.3 for the bead-beating method.

Droplet digital PCR is a novel technology that provides an absolute quantification of the target DNA with high precision, accuracy, and sensitivity. The DNA templates were isolated either with the QIAamp DNA mini kit or by the bead-beating method and diluted as presented on S1 Fig. Primer pairs used for *bla* and *dxs* amplification are listed in Table 1 (set A). Each measurement was done in two replicates (named bla1, bla2 and dxs1, dxs2; see Fig 1). The template DNA was obtained from two independent *E*. *coli* DH5α [pBR322] cultures (Experiment 1 and Experiment 2). The concentrations (copies/μl) of *bla* and *dxs* of total DNA isolated either with the QIAamp DNA mini kit or by the bead-beating method are shown on Fig 1A and 1B, respectively. The error reported for a single well was the Poisson 95% confidence interval. Representative 1D droplet plots show well defined clusters of *bla* and *dxs* positive droplets (blue colour) and cluster of droplets with no DNA (grey colour) (Fig 1C). Each droplet in the emulsion represents an independent nano-PCR. The threshold for positive and negative droplets and the concentrations of *bla* and *dxs* has been established automatically by the QuantaSoft droplet reader software. No primer-dimers were detected. The plasmid copy number is calculated by dividing the concentration of *bla* (copies μl^-1^) by *dxs* (copies μl^-1^). The plasmid copy numbers are presented on Fig 1D. The values for pBR322 vary between 19.85 and 21.73 copies per chromosome for the total DNA template isolated by the QIAamp DNA plasmid mini kit (average of 20.5), and between 6.92 and 7.49 copies per chromosome for the total DNA isolated by the bead-beating method (average of 7.3).

### Single colour, multiplex droplet digital PCR

**Fig 2:**
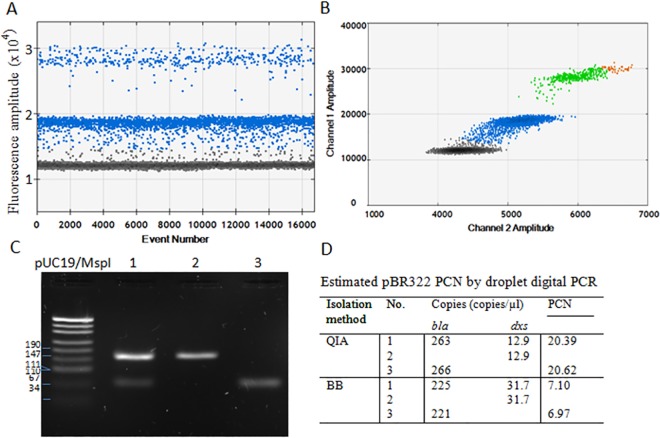
Quantification of pBR322 plasmid copy number with a single colour, multiplex, digital droplet PCR. (A) Column represents a single well of ~ 20,000 droplets containing *E*. *coli* DH5α total DNA template with multiplexed *bla* and *dxs*. (B) Representative droplet digital plot for PCR amplification of *bla* (blue colour), *dxs* (green colour) and double positive droplets (orange) in a multiplexed reaction for both targets. Grey colour represents droplets with no DNA. (C) *E*. *coli* DH5α total DNA served as a template for PCR amplification of *bla* (69 bp), *dxs* (160 bp), and a multiplexed reaction for both targets. PCR products were run in a 1.7% agarose gel. DNA marker- pUC19 DNA/MspI (HpaII) (Thermo Scientific). (D) Estimated pBR322 copy number by multiplex (tube no. 1) and single (tubes no. 2 and 3) droplet digital PCR. The plasmid copy number of pBR322 (PCN) was calculated by dividing the copy number of *bla* by the copy number of *dxs*.

The EvaGreen fluorescence is influenced by differences in the size of the amplicons [31]. The fluorescence amplitude of positive droplets increases with the amplicon length, as multiple dye molecules bind the same PCR product. Variation in the fluorescence signal intensity of EvaGreen, due to the size of DNA present in each droplet, was utilized for multiplexed detection. Optimal, 0.2 μM concentration of primers was also used, as primers concentration may influence the amplitude of droplets in EvaGreen based ddPCR [31]. We designed two pairs of primers which resulted in amplification of 69 bp and 160 bp DNA fragments for *bla* and *dxs*, respectively (Table 1B). Following amplification in the EvaGreen-containing reaction mixtures, positive droplets encapsulating the 160-bp DNA fragment (*dxs*) had higher fluorescence amplitude than those obtained for *bla* (Fig 2A). The 2D droplet plot revealed four different clusters of droplets: with no DNA (grey), *bla* positive (blue), *dxs* positive (green) and a small fraction of double positive droplets (orange) (Fig 2B). Manual thresholds were drawn to assign those clusters, but this factor did not significantly affect the results of the calculated DNA concentration. In parallel, we verified the presence of the two correctly sized PCR products by electrophoresis in a 1.7% agarose gel (Fig 2C). The table presented in Fig 2D shows the calculated plasmid copy number for each reaction carried in separate tubes (tubes no. 2 and 3) and for a multiplexed reaction (tube no. 1). This experiment was repeated with a DNA material isolated from an independent *E*. *coli* DH5α [pBR322] culture. The results indicated 247 copies μl^-1^ of *bla* and 36.6 copies μl^-1^ of *dxs*, which gave 6.7 copies of pBR322 for the BB DNA template isolation. Subsequently, for the QIAamp DNA mini kit template DNA extraction, we obtained 242 copies μl^-1^ of *bla* and 12 copies μl-1 of *dxs* which resulted in 20.17 copies of pBR322.

### Validation of droplet digital PCR method by real-time PCR

**Fig 3:**
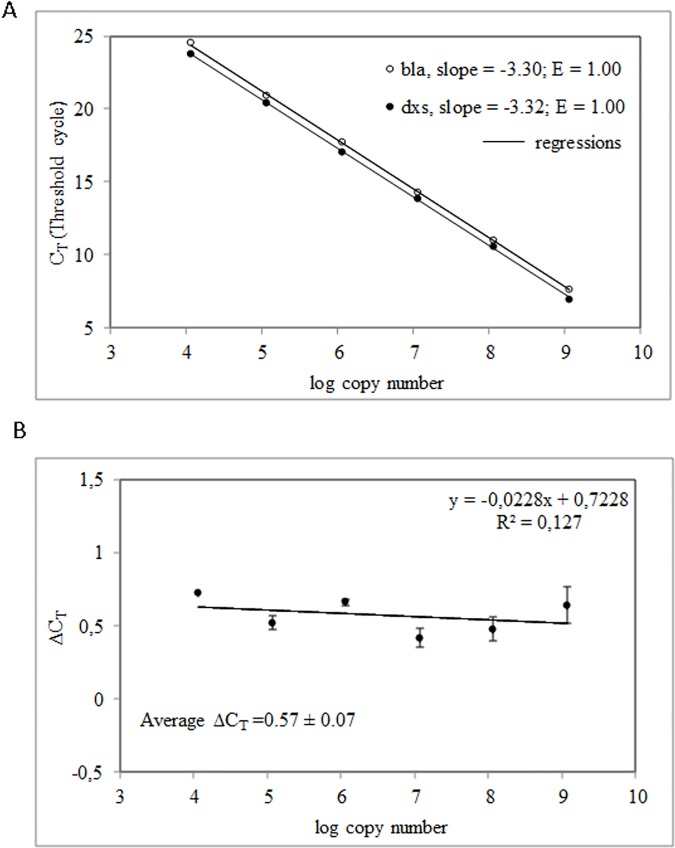
Construction of the standard curves for *bla* and *dxs*. (A) The standard curves were calculated with serial 10-fold dilutions of pGEM-dxs, ranging from 1 × 10^5^ to 1 × 10^9^ copies μl^-1^. Each standard dilution was amplified by qPCR using *bla* and *dxs* primer sets (*n* = 2). For each gene, the determined C_T_ values were plotted against the logarithm of their known initial copy number. A standard curve was generated by linear regression through these points. (B) Validation of the ΔΔC_T_ calculation. The ΔC_T_ deviation of *bla* vs. *dxs* was calculated for each dilution and plotted (*n* = 2). Average ΔC_T_ = average ± SD (*n* = 10).

To validate the droplet digital PCR methodology, we used real-time PCR as it became a "gold standard" in plasmid copy number determination. Total DNA was isolated from *E*. *coli* DH5α [pBR322] using QIAamp DNA Mini Kit (Qiagen). The chosen strain’s genetic background was *endA*ˉ because the presence of endonuclease A may negatively influence the quality of isolated DNA [32]. Non-optimized standard curves for *bla* and *dxs* are shown in S2 Fig. Both standard curves were linear, with average slopes of −3.32 and −3.54 for *bla* and *dxs* genes, respectively. Based on the standard curve slopes, amplification efficiencies of 1.0 and 0.92 were calculated for *bla* and *dxs*, respectively (S2 Fig). Because of differences in amplification efficiencies, the real-time PCR optimization was required. Different approaches, like supplementation of PCR reaction with organic compounds or genetic recombination proteins, have been used to improve the PCR efficiency and specificity [33, 34]. In our hands, we found that supplementation of a qPCR mixture with MgCl_2_ (3 mM) caused an increase in *dxs* amplification efficiency to E = 1.0 (Fig 3A). The experimentally defined C_T_ values correlated with the template dilutions. The fit of the model was satisfactory; the coefficient of determination (R^2^) was 0.999 for the triplicate reaction. A sensitive method for assessing if two amplicons have the same efficiency is to look at how ΔC_T_ varies with template dilution. Each of the 10-fold dilutions of a calibrator (pGEM-dxs) served as a template for amplification of *bla* and *dxs* genes in separate reactions. For three reaction replicates, the average C_T_ was calculated for both, *bla* and *dxs*, and the ΔC_T_ (C_T_ *bla* − C_T_ *dxs*) was determined. The plot in Fig 3B represents the log pGEM-dxs template dilution versus ΔC_T_. The slope of the lane is 0.0228 which proves that the efficiencies of the target and reference genes are similar, and the ΔΔC_T_ calculation for the relative quantification of *bla* may be used. Real time PCR amplifications of the *bla* and *dxs* genes from the total DNA isolated from three separate cultures of *E*. *coli* DH5α [pBR322] were performed simultaneously. The total DNA was isolated using QIAamp DNA Mini Kit (Qiagen). For absolute quantification, standard curves were generated with pGEM-dxs calibrator containing one copy of the *dxs* and *bla* specific sequences. The C_T_ values of each curve served for calculation of the absolute copy number of *bla* and *dxs* in the *E*. *coli* total DNA samples. The plasmid copy number of pBR322 was calculated by dividing the copy number of *bla* by the copy number of *dxs*. The total DNA samples from three independent cultures indicated similar plasmid copy numbers of 20.2, 22.7 and 21.7 (Table 2A). For relative quantification, the ΔC_T_ of the pGEM-dxs was given as 0.57 ± 0.07 by averaging the ΔC_T_ values determined from the 10-fold dilution series (Fig 3B and Table 2B). Because the amplification efficiency of both, *bla* and *dxs*, was 1.0, the plasmid copy number was determined by the 2^−ΔΔCT^ equation. The results obtained from three independent total DNA samples were 20.7, 23.4 and 22.5 copies per chromosome. Moreover, 10 fold dilution of the total DNA template at concentration of 0.2 ng/μl gave a similar pBR322 copy number to what was obtained for the undiluted sample: 20.2 *vs* 20.0 and 20.7 *vs* 20.3 for absolute and relative quantification, respectively (Table 2A and 2B, culture 1).

**Table 2:** Estimated plasmid copy number by (A) absolute and (B) relative quantification after DNA isolation by QIAamp DNA Mini kit.

A		Absolute quantification					
Culture		C_T_^a^			Copies^b^ (copies/μl)		PCN^b^
		*bla*		*dxs*	*bla*	*dxs*	
1	2 ng/μl	13.80 ± 0.03		17.60 ± 0.03	1.64 × 10^7^ (1.9%)	8.10 × 10^5^ (2.1%)	20.2 (0.1%)
	0.2 ng/μl	17.27 ± 0.03		21.04 ± 0.03	1.52 × 10^6^ (1.9%)	7.59 × 10^4^ (1.9%)	20.0 (0.1%)
2	2 ng/μl	13.76 ± 0.06		17.74 ± 0.06	1.68 × 10^7^ (3.8%)	7.40 × 10^5^ (4.4%)	22.7 (0.5%)
3	2 ng/μl	13.52 ± 0.03		17.44 ± 0.02	1.98 × 10^7^ (1.9%)	9.10 × 10^5^ (1.4%)	21.7 (3.4%)
B		Relative quantification					
Culture		C_t_^a^		ΔC_T_ sample	Calibrator^c^	ΔΔC_T_^a^	PCN^b^
		*bla*	*dxs*				
1	2 ng/μl	13.80 ± 0.03	17.60 ± 0.03	-3.80 ± 0.07	0.57 ± 0.07	-4.37 ± 0.07	20.7 (4.8%)
	0.2 ng/μl	17.27 ± 0.03	21.04 ± 0.03	-3.77 ± 0.00	0.57 ± 0.07	-4.34 ± 0.00	20.3 (0.0%)
2	2 ng/μl	13.76 ± 0.06	17.74 ± 0.06	-3.98 ± 0.06	0.57 ± 0.07	-4.55 ± 0.02	23.4 (2.4%)
3	2 ng/μl	13.52 ± 0.03	17.44 ± 0.02	-3.92 ± 0.09	0.57 ± 0.07	-4.49 ± 0.09	22.5 (5.4%)

^a^ Average ± SD (n = 3).

^b^ Average (coefficient of variation) (n = 3).

^c^ Calculated from the serial dilutions of the quantitative standard sample used for standard curve construction. Average ± SD (n = 10).

### The effect of DNA isolation method on plasmid copy number determination

**Fig 4:**
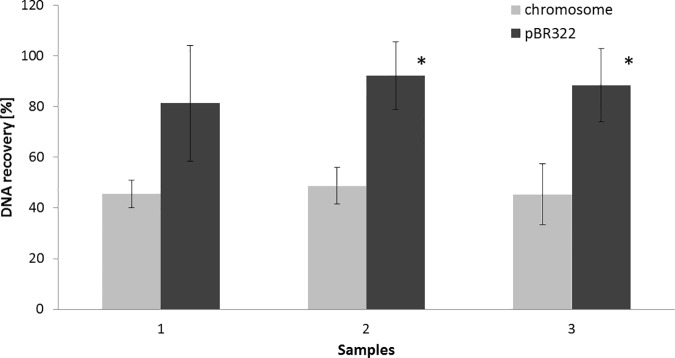
Efficiency of genomic and plasmid DNA recovery with the QIAamp DNA mini kit columns. Genomic and plasmid DNA were isolated from *E*. *coli* DH5α and *E*. *coli* DH5α [pBR322] with the use of Genomic and Plasmid DNA mini kits, respectively (A&A Biotechnology). DNA concentrations were measured by NanoDrop 1000 UV-VIS spectrophotometer (Thermo Scientific). Genomic DNA in the following amounts: 2110 ng, 1855 ng and 3922 ng, was coupled with 524 ng, 1100 ng and 1684 ng of plasmid DNA, respectively. Then, 100 μl of the lysis buffer (Qiagen) was added separately to genomic and plasmid DNA and the nucleic acids isolation was performed according to the QIAamp DNA mini kit manufacturer’s manual. The level of isolated DNA is indicated as a percentage relative to the unprocessed sample. The diagram represents three independent experiments. Error bars represent standard deviation (n = 3); (* *P*<0.02; Student *t* test).

The accuracy of PCN determination strongly relies on the precise ratio of chromosomal DNA to plasmid DNA [35–37]. Therefore, for the total DNA isolation from bacterial cells we decided to use a mechanical cell disruptor based on the bead-beating technology. A recent report on the comparative analysis of DNA extraction methods has shown a significant superiority of the mechanical cell disruption over other lysis procedures [38]. The method is based on disruption of cells by zirconia beads added to the suspension of cells. For plasmid copy number determination, 10-fold dilutions of the DNA template were used. The results of absolute quantification are shown in Table 3. The total DNA samples of pBR322 from three independent cultures, at logarithmic growth phase, showed similar plasmid copy numbers of 6.6, 6.9 and 7.2. However, these numbers were significantly lower than those calculated for DNA templates isolated by the QIAamp DNA mini kit. It is commonly known that the extraction protocols may suffer from inadequacies, including DNA retention on the mini column surfaces [39]. Therefore, we assessed the efficiency of chromosomal and plasmid DNA purification by the QIAamp DNA mini kit. Chromosomal and plasmid DNA, each at a known amount, were mixed separately with the lysis buffer and loaded onto the QIAamp DNA mini columns. The DNA recovery was calculated as percentage of the eluted DNA compared to the load. The result obtained showed that only 45.4% to 48.7% of the initial amount of *E*. *coli* chromosomal DNA was present in the eluate. The same applied to the plasmid DNA, where 81.37% to 92.27% of DNA was recovered (Fig 4). The statistical significance was marked by the asterisk on Fig 4, with *P*<0.02; Student *t* test.

**Table 3:** Estimated plasmid copy number by absolute quantification after DNA isolation by the bead-beating method.

Culture		C_T_^a^		Copies^b^ (copies/μl)		PCN^b^
		*bla*	*dxs*	*bla*	*dxs*	
LB OD_600_ = 0.5						
1	10^−1^	15.92 ± 0.05	18.09 ± 0.01	3.84 × 10^6^ (4.7%)	5.81 × 10^5^ (4.3%)	6.6 (6.2%)
	10^−2^	19.29 ± 0.02	21.52 ± 0.01	3.83 × 10^5^ (1.0%)	5.46 × 10^4^ (0.4%)	7.0 (4.5%)
2	10^−1^	17.05 ± 0.01	19.28 ± 0.05	1.78 × 10^6^ (0.5%)	2.55 × 10^5^ (3.2%)	6.9 (4.4%)
3	10^−1^	17.93 ± 0.01	20.21 ± 0.04	9.66 × 10^5^ (0.5%)	1.34 × 10^5^ (2.9%)	7.2 (2.4%)
LB OD_600_ = 1.5						
1	10^−1^	16.62 ± 0.04	19.79 ± 0.08	2.38 × 10^6^ (2.9%)	1.80 × 10^5^ (5.5%)	13.2 (1.0%)
	10^−2^	20.40 ± 0.19	23.76 ± 0.01	1.79 × 10^5^ (5.5%)	1.17 × 10^4^ (0.5%)	15.2 (5.9%)
2	10^−1^	17.02 ± 0.03	20.30 ± 0.04	1.80 × 10^6^ (2.1%)	1.27 × 10^5^ (2.8%)	14.7 (0.8%)
3	10^−1^	13.62 ± 0.02	17.05 ± 0.02	1.86 × 10^7^ (1.4%)	1.19 × 10^6^ (1.6%)	15.6 (1.2%)
TB OD_600_ = 0.5						
1	10^−1^	15.97 ± 0.02	18.16 ± 0.10	3.72 × 10^6^ (1.5%)	5.52 × 10^5^ (6.8%)	6.7 (5.4%)
	10^−2^	19.49 ± 0.04	21.58 ± 0.07	3.32 × 10^5^ (2.4%)	5.23 × 10^4^ (4.7%)	6.3 (2.4%)
2	10^−1^	17.23 ± 0.04	19.20 ± 0.04	1.57 × 10^6^ (3.4%)	2.70 × 10^5^ (3.0%)	5.8 (0.0%)
3	10^−1^	16.58 ± 0.02	18.76 ± 0.02	2.44 × 10^6^ (1.2%)	3.64 × 10^5^ (1.4%)	6.7 (2.6%)
M9 OD_600_ = 0.5						
1	10^−1^	16.29 ± 0.05	19.96 ± 0.08	2.99 × 10^6^ (3.4%)	1.59 × 10^5^ (5.2%)	18.8 (0.5%)
	10^−2^	19.97 ± 0.07	23.55 ± 0.06	2.40 × 10^5^ (4.7%)	1.34 × 10^4^ (3.9%)	17.4 (2.4%)
2	10^−1^	16.87 ± 0.03	19.63 ± 0.06	2.00 × 10^6^ (1.8%)	2.00 × 10^5^ (4.0%)	10.0 (4.8%)
3	10^−1^	15.64 ± 0.01	18.47 ± 0.05	4.64 × 10^6^ (0.4%)	4.45 × 10^5^ (3.5%)	10.4 (3.4%)

(*P* = 0.0651 for LB and TB; *P*<0.0001 for LB and M9 medium at OD_600_ = 0.5; Student *t* test).

^a^ Average ± SD (n = 3).

^b^ Average (coefficient of variation) (n = 3).

Because, the plasmid copy number changes in response to bacterial growth phase [40] and the culture medium employed [41], we cultivated bacteria in three different culture media: Luria-Bertani, Terrific broth and M9 minimal medium. The total DNA was isolated form *E*. *coli* DH5α cells by the bead-beating method. The plasmid copy number also varies in response to bacterial growth phase [40], therefore we isolated total DNA from bacteria in mid-logarithmic and stationary growth phase (LB, OD_600_ = 0.5 and OD_600_ = 1.5, respectively). The results obtained are shown in Table 3 (absolute quantification). The pBR322 copy number in LB when compared to terrific broth are similar (average 6.9 *vs* 6.4; *P* = 0.0651), and higher in bacterial stationary growth phase (6.9 *vs* 14.5; *P*<0.0001), as well as in the M9 minimal medium (6.9 vs 13.1; *P*<0.0001). These differences might be explained by the mechanism of plasmid DNA replication in *E*. *coli* DH5α during amino acid starvation (which is the case in stationary growth phase and during bacterial growth in M9 minimal medium). In the initiation process of *E*. *coli* plasmid DNA replication, a persistent hybrid is formed between the DNA template and the preprimer RNA II. The hybrid is necessary for subsequent primer generation that is elongated by the DNA polymerase I. On the other hand, the hybrid formation between RNA II and the DNA template might be blocked by the antisense RNA I molecule and in consequence no replication occurs. During amino acid starvation large amount of uncharged tRNA molecules are present in the bacterial cell. Uncharged tRNAs are able to bind both the RNA I or RNA II what prevents their interaction, but do not block the primer formation. Therefore, the uncharged tRNA abolishes the inhibitory role of RNA I and promotes the initiation of plasmid DNA replication what results in higher PCN in bacterial cells starved for amino acids [42]. The relative quantification calculations are shown in S1 Table and precisely reflect the absolute measurements.

## Discussion

To produce recombinant proteins or non-proteinous recombinant products appropriate expression system needs to be chosen. Among different parameters describing expression vectors, such as structural and segregational plasmid stability, plasmid copy number is an essential feature with strong impact on system productivity. In the present study, we developed a method for plasmid copy determination based on droplet digital PCR and EvaGreen, a next-generation DNA binding dye. This method is compatible with an approach that calculates the PCN parameter as a number of plasmid copies per chromosome [8]. However, there is also an alternative approach that estimates the PCN as a number of plasmid copies per cell [43]. It is important to stress that the results obtained with both approaches can differ. In the case of pBR322 present in fast growing bacteria (log phase), the number of plasmids per cell was calculated to be 39–55. However, in a parallel experiment the plasmid copy number per chromosome in the same phase of growth was estimated as 15–32 [44]. From those experiments it can be concluded that the average number of plasmid copies per cell is always higher than that calculated per chromosome. This phenomenon can be explained by multiple openings of the replication forks during bacterial exponential phase of growth that lead to the decrease of the plasmid per chromosome ratio and in consequence lower the plasmid copy number [18, 45]. Therefore, in the literature, depending on the methodology used, two different ranges of the pBR322 copy number exist: 15–20 [20] and 30–70 [46], and our results correspond to the lower PCN ranges. Apart from the different PCN calculation strategies (per chromosome or per cell), another source of variations in the PCN determination is the method used for DNA purification [37]. Real-time PCR requires only a small amount of the template DNA, but different efficiencies in the total DNA isolations can lead to the PCN miscalculation. Indeed, the most commonly used method for qPCR template purification is DNA isolation with the use of commercial kits or multi-step procedures involving cell disruption, often with the use of lysozyme, enzymatic protein digestion, DNA extraction with phenol-chloroform, precipitation and rehydratation [13, 20, 43]. However, it is already known that each step added to the DNA purification procedure increases the probability of the sample loss [37]. Therefore, for efficient DNA isolation, we replaced the multi-step DNA isolation procedure by a simple, mechanical disruption of bacterial cells by the bead-beating method. In our hands, the bead-beating seemed to be a simple and fast strategy to isolate total DNA that was ready to be used in the qPCR experiments. In recent years, the bead-beating method was successfully used for efficient DNA isolation for various applications [47–49]. For example, among five different mechanical cell disruption methods, including sonication, nebulization, homogenization, microfluidization, and bead milling (bead-beating; BB in this paper), the bead milling was found to be the most efficient for intact plasmid extraction from bacterial cells, with the recovery yield reaching over 90% [47]. Moreover, it was shown that only bead-beating was effective for isolating DNA from such difficult samples as *Bacillus globigii* (*B*. *subtilis* subsp. *niger*) endospores or *Fusarium moniliforme* conidia [48]. In our hands, two different methods used for DNA isolation gave two different ranges of the pBR322 copy number (Tables 2 and 3 and S1 Table). For the *E*. *coli* bacteria in the log-phase, the pBR322 PCN was in a range of 6–7 for the bead-beating method, and 20–23 for the QIAamp DNA Mini kit used for DNA isolation. It is a common knowledge that there is a difference in efficiency of isolation of plasmid or chromosomal DNA, especially when the DNA binding columns are used. We have shown that in defined experimental settings, when mixture of genomic and plasmid DNA was loaded onto a purification column, on average only 46.54% of the initial amount of *E*. *coli* chromosomal DNA was present in the eluate. The same applied to as much as 87.38% of the plasmid DNA (Fig 4). This difference may distort the ratio of the plasmid to chromosomal DNA and lead to over-estimation of the plasmid copy number. Problems with the multi-step DNA isolation procedures were also noticed by other scientists [18, 37, 43]. In many laboratories, the researchers had started to prepare DNA for the PCN determination by heating the cell samples at 95°C [43], and different heating protocols were tested for an optimal template preparation [18, 37]. In our project, we performed the direct comparison between a multi-step procedure and a simple, mechanical cells disruption method for DNA extraction. Similar analyses had not been performed by others so far.

Besides using different DNA isolation methods to calculate the pBR322 copy number, we successfully applied DNA endogenous controls independent, droplet digital PCR technology to evaluate the obtained results. The ddPCR methodology does not rely on the use of standard curves for absolute quantification of nucleic acids. The ddPCR is also less susceptible to PCR inhibitors often co-extracted with nucleic acids from environmental samples and shows better reproducibility at low target concentrations than qPCR [37, 50]. Moreover, the method demonstrates improved detection of low copy number DNA target, because the large-scale partitioning involved with ddPCR removes the potential competition with extraneous DNA targets for primers or other reagents [23]. Droplet digital PCR has been used before to calculate the plasmid copy number of *Pseudomonas putida* KT2440 plasmid pA-EGFP_B [37] or a set of six certified reference plasmid solutions (ERM-AD623a–f) from the Joint Research Centre-Institute for Reference Materials and Measurements (JRC-IRMM, European Commission, Belgium) [51]. However, in both cases specific, dual colour, oligonucleotide probe dependent ddPCR was performed. Oligonucleotide probe dependent ddPCR technique requires optimization of the particular reaction and the optimized reaction cannot be adapted to evaluate copy number of unrelated plasmid. In contrast to oligonucleotide probe dependent ddPCR, the EvaGreen single colour ddPCR designed in this study can be more widely used. Obtained ddPCR results fully confirmed the absolute and relative qPCR calculations. With the use of ddPCR, we also observed the same dependency of PCN on the total DNA isolation method used. Moreover, we show a possibility of performing the detection of both, the target (*bla*) and the reference (*dxs*) genes, in a single tube by differentiating the amplicon length (Fig 2). ddPCR is a simple, straightforward method for PCN calculation. It allows performing thousands of PCR reactions in a single tube which significantly increases the precision of PCN calculations. Therefore, we propose ddPCR as the preferred method of choice for determination of plasmid copy number.

## Conclusions

To our knowledge, for the first time, we applied a single colour ddPCR (with the use of EvaGreen) for determination of the plasmid copy number. Because, the β-lactamase is a common selection marker present in many expression systems, and *E*. *coli* is a popular bacterial host, we believe that ddPCR method developed in this study can be easily adopted by other researches to evaluate plasmid copy number. Moreover, with a little effort the designed method can be optimized to be used for other selection markers (such as genes encoding resistance to chloramphenicol or tetracycline) and different bacterial hosts.

## Supporting Information

S1 Fig Optimization of the amount of DNA template used for ddPCR. (PDF)

S2 Fig Non-optimized standard curves for *bla* and *dxs*. (PDF)

S1 Table Estimated plasmid copy number by relative quantification after DNA isolation by the bead-beating method. (PDF)
