# Supplementary material for: Quantification of Plasmid Copy Number with Single Colour Droplet Digital PCR
Source: PLoS One. 2017 Jan 13;12(1):e0169846. doi: 10.1371/journal.pone.0169846 (PMC5234771; doi:10.1371/journal.pone.0169846)
Supplement: S1 Fig — (PDF) [file pone.0169846.s001.pdf]

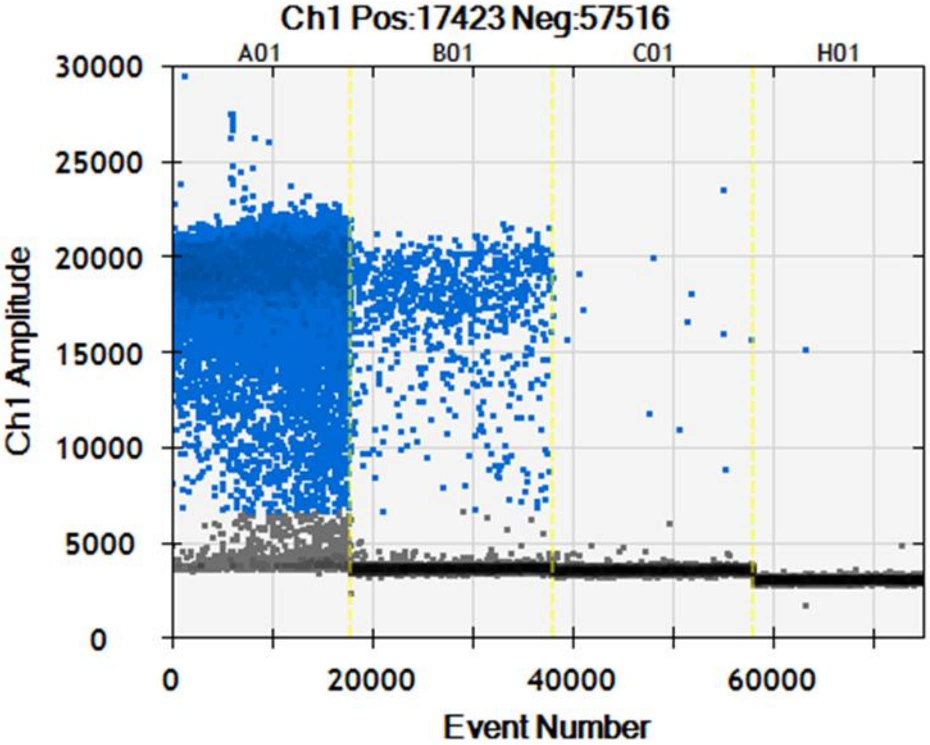

Figure S1. Optimization of the amount of DNA template used for ddPCR. Columns represent single wells of ~20,000 droplets after ddPCR amplification of *dxs* gene with use of primer set A (Table 1). The DNA template was isolated with the QIAamp DNA mini kit from *E. coli* DH5α [pBR322]. The DNA template was diluted as follows: A01 – 10 x; B01 – 1000 x; C01 - 100,000 x; H01 – no template control. The DNA template 1000 x diluted has been chosen for further analysis (B01).
