# Supplementary material for: Quantification of Plasmid Copy Number with Single Colour Droplet Digital PCR
Source: PLoS One. 2017 Jan 13;12(1):e0169846. doi: 10.1371/journal.pone.0169846 (PMC5234771; doi:10.1371/journal.pone.0169846)
Supplement: S2 Fig — (PDF) [file pone.0169846.s002.pdf]

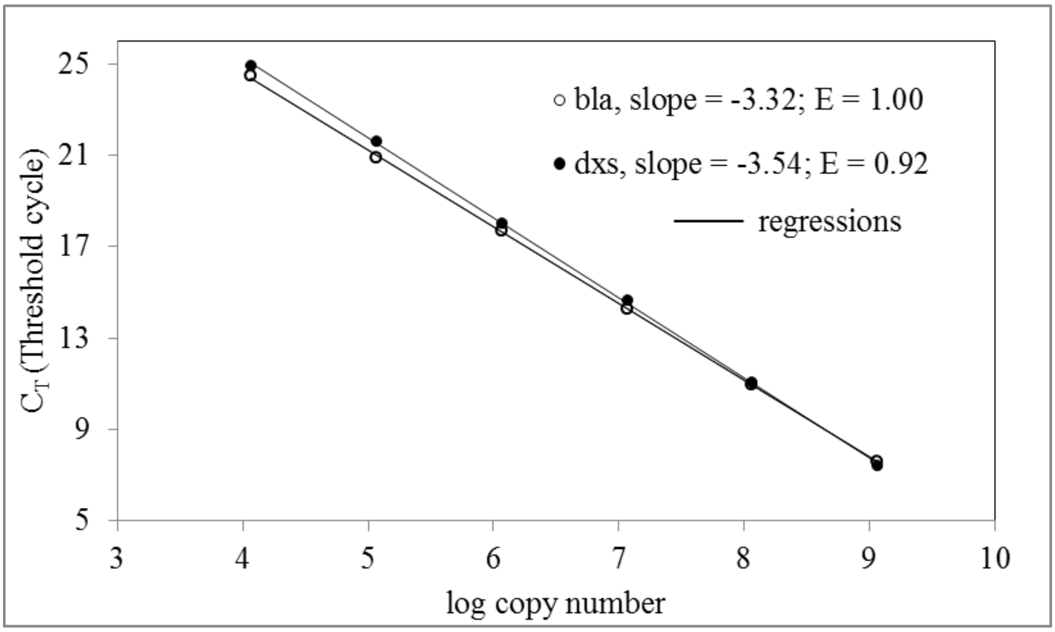

Figure S2. Non-optimized standard curves for *bla* and *dxs*. The standard curves were calculated with serial 10-fold dilutions of pGEM-dxs, ranging from 1 x 10^5 to 1x 10^9 copies μl^{-1}. Each standard dilution was amplified by qPCR using *bla* and *dxs* primer sets (*n* = 2). For each gene, the determined C_T values were plotted against the logarithm of their known initial copy number. A standard curve was generated by linear regression through these points.
