# Supplementary material for: Quantification of Plasmid Copy Number with Single Colour Droplet Digital PCR
Source: PLoS One. 2017 Jan 13;12(1):e0169846. doi: 10.1371/journal.pone.0169846 (PMC5234771; doi:10.1371/journal.pone.0169846)
Supplement: S1 Table — (PDF) [file pone.0169846.s003.pdf]

Table S1. Estimated plasmid copy number by relative quantification after DNA isolation by the bead-beating method.

Culture	C_t[a]		ΔC_T sample	Calibrator[b]	$\Delta\Delta C_T$[a]	PCN[c]
	bla	*dxs*				
LB OD_{600}=0.5						
1 10^{-1}	15.92 ± 0.05	18.09 ± 0.01	-2.17 ± 0.05	0.57 ± 0.07	-2.74 ± 0.05	6.7 (5.4%)
10^{-2}	19.29 ± 0.02	21.52 ± 0.01	-2.23 ± 0.06	0.57 ± 0.07	-2.80 ± 0.04	7.0 (5.3%)
2 10^{-1}	17.05 ± 0.01	19.28 ± 0.05	-2.23 ± 0.06	0.57 ± 0.07	-2.80 ± 0.06	7.0 (4.4%)
3 10^{-1}	17.93 ± 0.01	20.21 ± 0.04	-2.28 ± 0.04	0.57 ± 0.07	-2.85 ± 0.04	7.2 (2.5%)
LB OD_{600}=1.5						
1 10^{-1}	16.62 ± 0.04	19.79 ± 0.08	-3.17 ± 0.06	0.57 ± 0.07	-3.74 ± 0.09	13.4 (7.3%)
10^{-2}	20.40 ± 0.19	23.76 ± 0.01	-3.36 ± 0.01	0.57 ± 0.07	-3.93± 0.01	15.2 (0.5%)
2 10^{-1}	17.02 ± 0.03	20.30 ± 0.04	-3.28 ± 0.01	0.57 ± 0.07	-3.85 ± 0.01	14.4 (0.8%)
3 10^{-1}	13.62 ± 0.02	17.05 ± 0.02	-3.43 ± 0.02	0.57 ± 0.07	-4.00 ± 0.02	16.0 (1.2%)
TB OD_{600}=0.5						
1 10^{-1}	15.97 ± 0.02	18.16 ± 0.10	-2.19 ± 0.24	0.57 ± 0.07	-2.76 ± 0.24	6.8 (24.9%)
10^{-2}	19.49 ± 0.04	21.58 ± 0.07	-2.09 ± 0.08	0.57 ± 0.07	-2.66 ± 0.08	6.3 (5.1%)
2 10^{-1}	17.23 ± 0.04	19.20 ± 0.04	-1.97 ± 0.01	0.57 ± 0.07	-2.54 ± 0.01	5.8 (0.5%)
3 10^{-1}	16.58 ± 0.02	18.76 ± 0.02	-2.18 ± 0.04	0.57 ± 0.07	-2.75 ± 0.04	6.7 (2.3%)
M9 OD_{600}=0.5						
1 10^{-1}	16.29 ± 0.05	19.96 ± 0.08	-3.67 ± 0.08	0.57 ± 0.07	-4.24 ± 0.09	18.9 (7.3%)
10^{-2}	19.97 ± 0.07	23.55 ± 0.06	-3.58 ± 0.08	0.57 ± 0.07	-4.15 ± 0.08	17.8 (7.4%)
2 10^{-1}	16.87 ± 0.03	19.63 ± 0.06	-2.76 ± 0.04	0.57 ± 0.07	-3.33 ± 0.04	10.1 (2.5%)
3 10^{-1}	15.64 ± 0.01	18.47 ± 0.05	-2.83 ± 0.03	0.57 ± 0.07	-3.40 ± 0.04	10.6 (3.5%)

[a] Average ± SD (n = 3).
[b] Calculated from the serial dilutions of the quantitative standard sample used for standard curve construction. Average ± SD (n = 10).
[c] Average (coefficient of variation) (n = 3).
